# Radiotherapy planning parameters correlate with changes in the peripheral immune status of patients undergoing curative radiotherapy for localized prostate cancer

**DOI:** 10.1007/s00262-021-03002-6

**Published:** 2021-07-16

**Authors:** Elgin Hoffmann, Frank Paulsen, Philipp Schaedle, Daniel Zips, Cihan Gani, Hans-Georg Rammensee, Cécile Gouttefangeas, Franziska Eckert

**Affiliations:** 1 Department of Radiation Oncology, University Hospital Tuebingen, Eberhard-Karls-University Tuebingen, Hoppe-Seyler-Str. 3, 72076 Tuebingen, Germany; 2 Interfaculty Institute for Cell Biology, Department of Immunology, Eberhard-Karls-University Tuebingen, Auf der Morgenstelle 15, 72076 Tuebingen, Germany; 3 German Cancer Consortium (DKTK), German Cancer Research Center (DKFZ) Partner Site Tuebingen, Tuebingen, Germany; 4 Present Address: Department for Internal Medicine I, Marienhospital Stuttgart, Boeheimstr. 37, 70199 Stuttgart, Germany; 5 Cluster of Excellence iFIT (EXC2180) ‘Image-Guided and Functionally Instructed Tumor Therapies’, University of Tuebingen, Tuebingen, Germany

**Keywords:** Prostate cancer, Localized, IMRT, DVH, Immune status, T cells

## Abstract

**Purpose:**

The influence of radiotherapy on patient immune cell subsets has been established by several groups. Following a previously published analysis of immune changes during and after curative radiotherapy for prostate cancer, this analysis focused on describing correlations of changes of immune cell subsets with radiation treatment parameters.

**Patients and methods:**

For 13 patients treated in a prospective trial with radiotherapy to the prostate region (primary analysis) and five patients treated with radiotherapy to prostate and pelvic nodal regions (exploratory analysis), already published immune monitoring data were correlated with clinical data as well as radiation planning parameters such as clinical target volume (CTV) and volumes receiving 20 Gy (V20) for newly contoured volumes of pelvic blood vessels and bone marrow.

**Results:**

Most significant changes among immune cell subsets were observed at the end of radiotherapy. In contrast, correlations of age and CD8^+^ subsets (effector and memory cells) were observed early during and 3 months after radiotherapy. Ratios of T cells and T cell proliferation compared to baseline correlated with CTV. Early changes in regulatory T cells (Treg cells) and CD8^+^ effector T cells correlated with V20 of blood vessels and bone volumes.

**Conclusions:**

Patient age as well as radiotherapy planning parameters correlated with immune changes during radiotherapy. Larger irradiated volumes seem to correlate with early suppression of anti-cancer immunity. For immune cell analysis during normofractionated radiotherapy and correlations with treatment planning parameters, different time points should be looked at in future projects.

**Trial registration number:**

NCT01376674, 20.06.2011

**Supplementary Information:**

The online version contains supplementary material available at 10.1007/s00262-021-03002-6.

## Introduction

Radiotherapy, mostly in combination with androgen deprivation therapy (ADT), is one of the curative options for localized prostate adenocarcinoma. Depending on the risk classification, tumor control rates of > 85%, > 75% and > 55% for low, intermediate and high-risk prostate cancer patients can be achieved after radiotherapy with 74 Gy [1]. With image-guidance and conformal radiotherapy planning, higher radiation doses have become achievable without excessive toxicity [2].

Although not established in clinical routine for the treatment of localized prostate cancer [3], immunotherapeutical approaches have become a major player in oncology in general [4], including for metastatic prostate cancer [5]. Several clinical trials have been published and are ongoing [6, 7]. Combining immunotherapy with radiotherapy has become a major field of investigation [8–10]. However, there are a lot of uncertainties about the specifics of radiotherapy (dose, fractionation, timing) to be best combined with immunotherapy. Sequencing of different therapy modalities, as well as fractionation and dosing of radiotherapy, seems to play a major role for synergistic effects [11]. For the combination with PD1 / PD-L1 blockade, simultaneous application has been described as favorable in preclinical models [12]. In contrast, the effect of adoptive T cell transfer was most pronounced when given at late time points after radiotherapy [13]. There is a strong rationale for hypofractionated irradiation in combination with CTLA4 blockade [14]. Optimal sequencing, fractionation and total doses will probably vary for the immunotherapy modality applied [15, 16].

Immune biomarker assessment in patients undergoing cancer therapy should generate helpful data for determination of which time points in standard cancer therapies might be best for combination with different immunotherapy approaches and which clinical settings and treatment schedules might be most promising. Multiparameter flow cytometry is the method of choice for analyzing immune cell subsets in peripheral blood over time [17, 18]. Moreover, analysis of changes in subsets of immune cells during standard cancer treatments might help in finding the optimal time point and scheduling for future treatment options. Examples for informative reports are studies which observed that definitive radiochemotherapy for cervical cancer led to profound immunosuppression which might limit the advantage of combined approaches with immunotherapy [19]. For patients undergoing surgery for lung cancer, several blood immune cell subsets showed prognostic significance [20]. Our own data, on which this report is based, have shown significant changes in peripheral immune cell subsets in prostate cancer patients undergoing definitive radiotherapy (± androgen deprivation), not all of which had subsided three months after end of treatment [21].

The relationship between radiation dose to the pelvic bone marrow and hematologic toxicity, mostly leukopenia and lymphopenia, has been described for radiochemotherapy of cervical [22] and anal cancers [23]. For radiotherapy of prostate cancer patients, long-lasting lymphopenia was observed, especially for prescribed higher doses to nodal volumes and for older patients [24]. Lymphopenia was also related to the radiation dose to pelvic bone marrow as described by Sini et al. [25].

Cell subsets analyzed in this study comprise general categories such as T cells and B cells, but also functionally characterized subsets. Effector T cells are cells directly involved in anti-cancer immune responses [26]. Memory T cells play a crucial role in long-lasting immunity and protection against relapses of cancer after curative treatment [27]. Treg cells are the subset responsible for ending overwhelming immune responses after infections. In cancer immunology, they are well known for negatively impacting anti-cancer immunity [28].

The aim of this study was to relate our already published immunomonitoring data for prostate cancer patients undergoing definitive radiotherapy [21] with radiation planning parameters (target volume, treated volume of immune-associated structures such as pelvic bone marrow and large pelvic blood vessels). With the in-depth immunomonitoring data acquired during this study, a detailed description of immunologic changes is possible.

## Patients and methods

### Patients and treatment

In a prospective study, peripheral blood mononuclear cells (PBMCs) from 18 of 63 initially screened patients were collected during and after curative radiotherapy with (n = 16) or without (n = 2) androgen deprivation therapy in the years 2011 and 2012. The study was approved by the local ethics committee (project number 402/2010BO2) and registered at www.clinicaltrials.gov (NCT01376674). The results describing longitudinal assessments of various immune cell subsets were published in 2018 [21]. Patients were treated with 70–78 Gy in 35 to 39 fractions to the prostate with base of seminal vesicles in case of stage T3a or less or to the prostate with entire seminal vesicles in case of T3b stage, respectively, according to institutional standards at that time. Of the 18 patients included in this analysis (median age of 75 years, range 68–82 years), five were also treated with 50.4 Gy to the pelvic nodal regions, offered to patients with high-risk prostate cancer. Radiotherapy was planned based on three planning CTs using a coverage probability approach as published previously [29, 30]. Due to large differences in dose levels to pelvic areas for patients treated with elective nodal irradiation, the primary analysis was focused on the patient cohort undergoing radiotherapy to prostate with / without seminal vesicles only. The five patients treated with elective nodal irradiation were analyzed separately in an exploratory analysis.

### Immune cell subsets

Peripheral immune cell subsets were characterized by multiparameter flow cytometry as described in detail previously [21]. Blood samples were obtained before start of treatment (time point A), twice during therapy at 1-month intervals (time points B, C) and three months after the end of treatment at a follow-up visit (time point D). Sample size for cell sub-populations analyzed at each time point is specified in Suppl. Table 1. PBMCs were isolated by density gradient centrifugation and frozen until use. All PBMCs from each patient were analyzed in the same experiment. T cells were defined as CD3^+^CD19^−^ lymphocytes and further characterized as CD4^+^ or CD8^+^. Regulatory T cells were identified as CD4^+^CD25^+^FoxP3^+^ T cells, B cells as CD3^−^CD19^+^ and natural killer cells (NK) as CD3^−^CD19^−^ lymphocytes. Proliferation status (Ki67^+^) was assessed for all of these subsets. Naïve T cells were defined as CD45RA^+^CD28^+^, effector cells as CD45RA^+^CD28^−^. For every immune cell subset, intra-individual ratios of the percentages at timepoints B, C and D were calculated referring to baseline (time point A).

### Analysis of radiotherapy planning parameters

All radiation treatment plans were calculated with Hyperion®, a Monte-Carlo-based treatment planning system using an EUD (equivalent uniform dose)-based optimization concept. All treatments were planned as static IMRT using 8 gantry angles in 17 cases, 5 gantry angles in one case. All radiation treatment plans were reviewed, and dose and volume parameters were recorded for clinical target volume (CTV) and planning target volume (PTV).

### Contouring of pelvic bone marrow and vessels

For all patients, additional volumes with a possible relationship to immune parameters were contoured, namely bone marrow, pelvic and iliac lymph nodes and large vessels. Pelvic bone marrow, as well as pelvic bones, was contoured as iliac left and right, lumbosacral and lower pelvic volumes for two patients. Dose parameters were compared for bone marrow and bone structures, respectively. Further analysis was performed with bone structures. The hull was termed pelvic bone marrow union (PBM union) following the contouring as suggested by Li et al. [31]. In addition, large pelvic blood vessels were contoured, with craniocaudal borders defined by the bone marrow contour borders, including aorta and external iliac and inguinal arteries and veins, the hull being termed vessels. Dose-volume histograms (DVH) for the original, clinically used treatment plans, were extracted from the planning system, and percentage of volume receiving X Gy or more (VX; X = 10, 20, 30, 40 and 50) were recorded for every volume. For further analysis, V20 values were used for contoured blood vessels as well as total pelvic bone marrow (PBM). V20 was chosen as it was the highest dose level with values > 0cm^3^ in all patients. Fraction dose for V20 in 35–37 fractions is 0.54–0.57 Gy and thus a range with a predicted biological effect, corresponding to the dose with a 90% survival fraction of lymphocytes [32]. For PBM union correlation of V10, V30, V40 and V50 were analyzed additionally. For large blood vessels analysis of V10 and V30 was performed as well.

### Statistical analysis

The statistical analysis was done with GraphPad 8.4.0 (GraphPad, San Diego, CA, the USA) and SPSS24 (SPSS Inc., Chicago, IL, the USA). Correlations of linear parameters were characterized by the Pearson correlation coefficient (r). Moderate and strong correlations were defined by Pearson correlation coefficients of 0.4–0.7 and > 0.7, respectively. Means were compared by student’s t test if the assumptions for the test were met and Bonferroni corrected in case of multiple testing. P values were considered significant if < 0.05, with Bonferroni correction zp < 0.05 (z = number of comparisons). Means are given ± standard error of the mean.

## Results

### Immune cell ratios compared to baseline

**Fig. 1:**
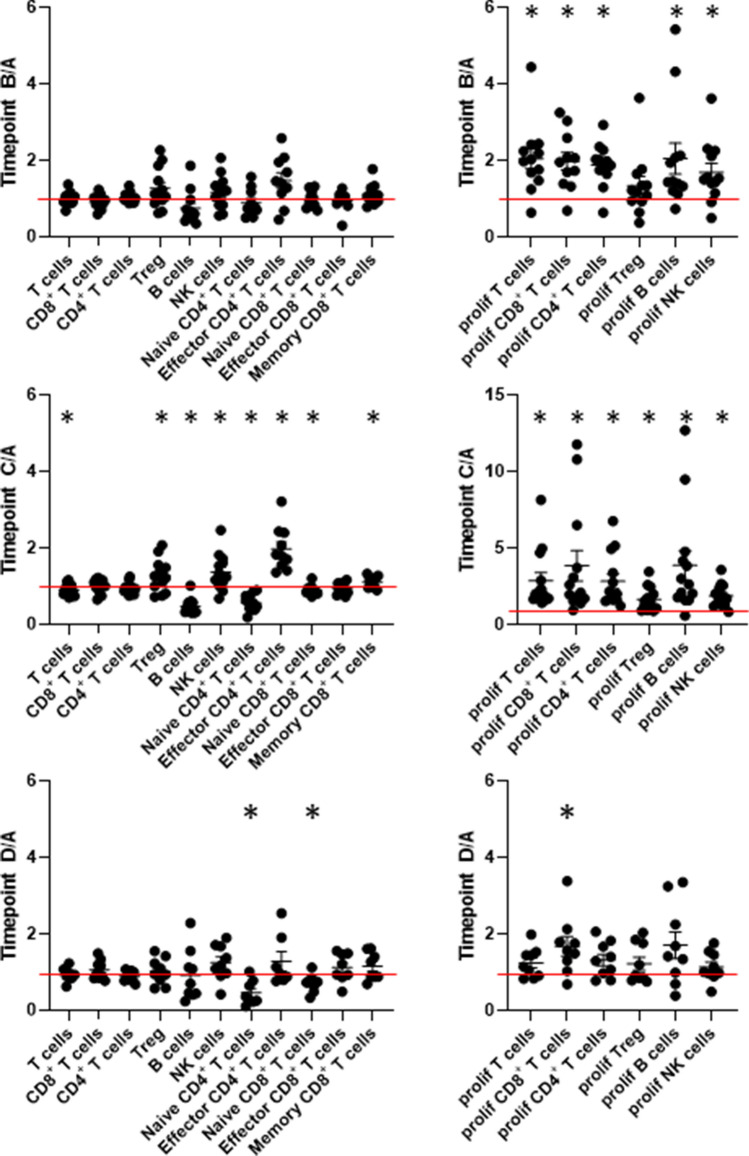
To compare immune cell changes with radiation planning parameters, the ratio of immune cell subsets and proliferative fractions within each of these cell subsets is displayed for time point B (4 weeks into radiotherapy), time point C (end of radiotherapy) and time point D (3 months after end of radiotherapy) compared to baseline levels (time point A). Significant changes have been observed for proliferation of most cell subsets at time point B, most subsets at time point C, and only a few long-lasting effects at time point D. The red line indicates a ratio of 1 and thus no change compared to baseline frequencies. * indicates significant changes with one sample t-test compared to a ratio of 1

In order to correlate immune cell changes during radiotherapy with radiation planning parameters, intra-individual ratios of immune cell subsets were calculated for each time point during or post-radiotherapy (time point B (4 weeks into radiotherapy), time point C (end of treatment) and time point D (first follow-up after three months)) compared to before therapy (time point A) as shown in Fig. 1. The values used for ratio calculation, which were % of the cell subsets of interest, have been reported in a previous publication [21]. Significant early changes at time point B were limited to increased proliferation of most tested cell subsets except for Tregs (Fig. 1, upper panel). Percentages of cell numbers did not show significant alterations. Most significant changes in immune cell subsets, as well as in proliferation, were observed comparing time point C to time point A (Fig. 1, middle panel). T cells and B cells were significantly decreased, while NK and Treg cell frequencies were significantly higher compared to baseline. In the CD4^+^ subset, effector cells were more prominent, and inversely naïve cells were decreased. All cell subsets showed a significantly increased proliferation. Three months after end of the radiotherapy, most immune cell changes had recovered with the exception of low naïve CD4^+^ and CD8^+^ T cells, and an enhanced proliferation rate of whole CD8^+^ T cells (Fig. 1, time point D to time point A, lower panel). As two patients were treated without ADT, we plotted the data of these patients separately in comparison to the eleven patients with bimodal treatment. The small number of patients did not allow for statistical analysis, but no marked differences were detected (data not shown). Thus, for all further analyses, these two patients were included.

### Immune cell ratios and patient age

**Fig. 2:**
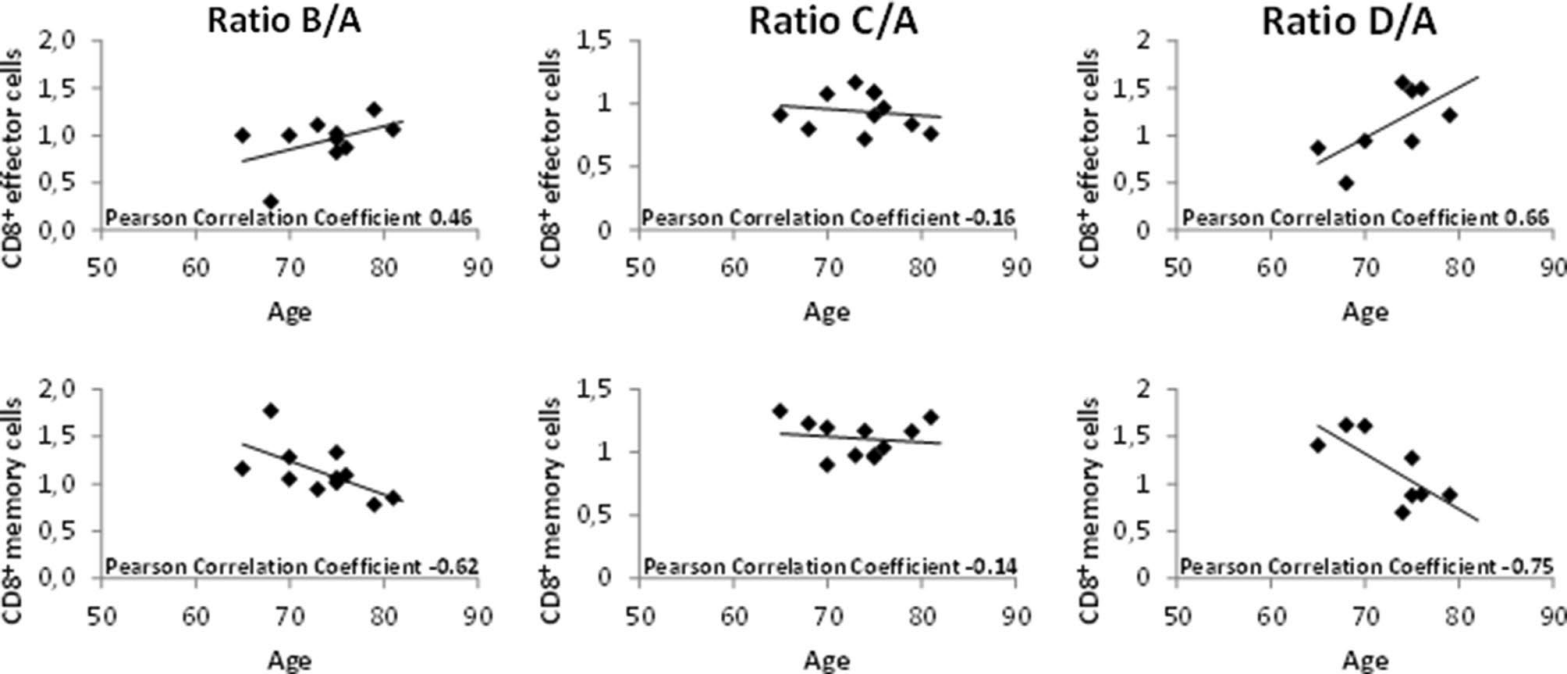
Correlation of patient age at time of radiotherapy and CD8^+^ effector cells as well as CD8^+^ memory cells is shown for all time points in the subgroup of patients treated with radiotherapy to the prostate. Patient age correlated positively with CD8^+^ effector cells and negatively with CD8^+^ memory cells at early and late time points (B/A and D/A, respectively)

Considering the impact of aging on the immune system, we next asked whether the changes in immune cell subsets were impacted by the age of the patients. Ratios at the earliest time during radiotherapy (time point B) showed moderate or strong correlations with patient age at start of treatment. Among other correlations (for details, refer to Suppl. Table 2, listing all Pearson correlation coefficients), a positive correlation for CD8^+^ effector cells and a negative correlation for CD8^+^ memory cells were observed (ratio B/A, Fig. 2 left panel). These were lost at a later time point during therapy (ratio C/A, Fig. 2. middle panel), but again observed during the recovery phase 3 months after treatment (ratio D/A, Fig. 2, right panel). Percentage of CD8^+^ effector cells and CD8^+^ memory cells at time points A, B, C and D considered separately did not correlate with patient age (data not shown). Only one moderate positive correlation was found at the end of radiotherapy for CD4^+^ effector cells and patient age (data not shown). These results were not confirmed in the exploratory cohort (data not shown).

### Contouring of lymphocyte rich structures

**Fig. 3:**
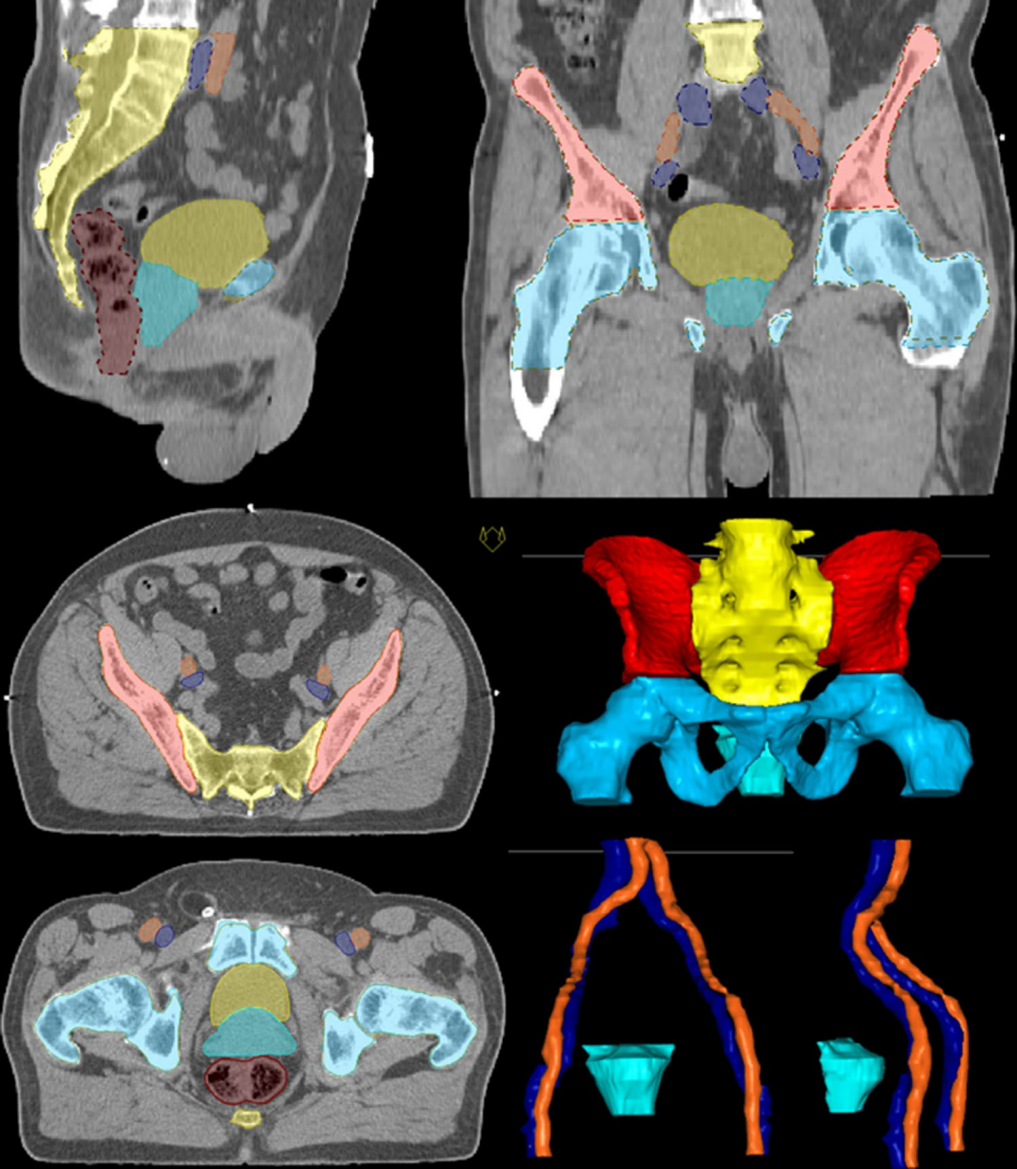
Volumes contoured in addition to standard radiotherapy target volume (turquoise) and organs at risk such as bladder (yellow) and rectum (brown) are pelvic bone marrow (PBM union) comprising iliac bone marrow (red), lumbosacral spine (yellow) and lower pelvic bone marrow (light blue) as well as arteries (orange) and veins (dark blue) combined as vessels are shown

In order to compare intra-individual ratios of immune cell subsets with radiation planning parameters beyond the size of target volumes, lymphocyte-rich structures were contoured for every treatment plan, and DVH parameters of the clinically applied radiation plans were extracted. The pelvic bones were contoured in analogy to Li et al. [31]. For two patients, pelvic bones as well as pelvic bone marrow were contoured in order to compare DVH parameters for both volumes. DVH parameters for iliac bone marrow, lower pelvic bone marrow and lumbosacral bone marrow were compared (V10, V20, V30, V40, V50) to corresponding data for the whole bones including calcified regions, and no significant difference was detected (comparison of dose parameters for 6 volumes, data not shown). As a consequence, for all other patients (*n* = 16), only pelvic bones as a whole were contoured. The respective contoured volumes for all patients were 266.1 ± 8.1 cm^3^, 448.3 ± 18.1 cm^3^, 723.3 ± 22.7 cm^3^ for iliac bone marrow, lumbosacral bone marrow and lower pelvic bone marrow, respectively. The analysis was performed with V20 of the pelvic bone marrow (hull of iliac bone marrow, lower pelvic bone marrow and lumbosacral bone marrow, PBM union) with a mean volume of 1708.1 ± 46.2 cm^3^. In order to compare radiation doses to the blood vessels to immune cell subsets in the peripheral blood, large pelvic blood vessels were contoured as well. These include the external iliac vessels (arteries and veins) starting at the bifurcation, reaching to inguinal and femoral region. Contoured volume was 193.1 ± 8.3 cm^3^. For these volumes analysis was also performed with V20 values. Contoured volumes and CTV, rectum, bladder and vessels are shown for one patient in Fig. 3.

**Fig. 4:**
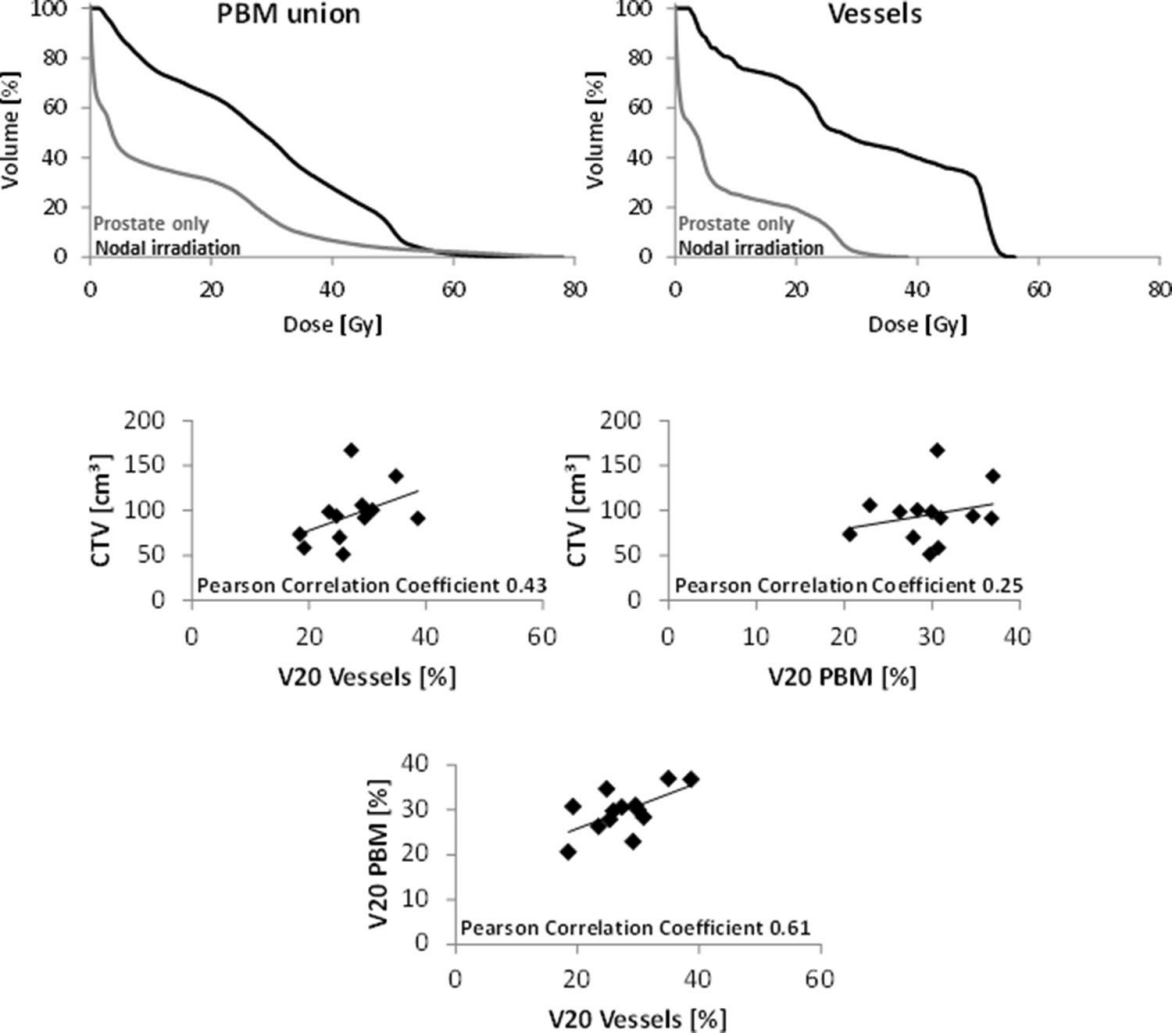
Dose-volume histograms of PBM union and vessels (upper panels) are shown for two patients comparing radiotherapy to the prostate only and radiotherapy including pelvic node irradiation. Correlations of CTV and V20 of vessels and PBM are shown for all patients. The clinical target volume (CTV) used for radiation planning and dose parameters for PBM and vessels showed no to moderate correlation to each other. Thus, the volume of CTV is not an adequate surrogate parameter for the dose to PBM and vessels (V20). V20 for PBM and V20 for vessels showed a moderate correlation

Dose-volume histograms of the five patients treated to pelvic node irradiation show a marked difference in dose levels to PBM union and vessels compared to patients treated to prostate volumes only (Fig. 4, upper panels). Thus, pooling all patients for analysis with immune parameters is not reasonable. Primary analysis was limited to the patients, who received radiotherapy to the prostate only. The five patients treated with radiotherapy to elective nodal regions were used for an exploratory analysis.

### Interdependence of CTV, V20 (PBM union) and V20 (vessels)

In order to select parameters for comparison with immune cell subset changes, the interdependence of CTV, V20 (PBM union) and V20 (vessels) was determined. Whereas a Pearson correlation coefficient of 0.61 indicates a moderate correlation between V20 (vessels) and V20 (PBM union) (Fig. 4, lower panel), V20 (vessels) and V20 (PBM union) showed no or only moderate correlations with the CTV (Fig. 4, second row panels). Thus, all three parameters were considered for subsequent analysis in conjunction with immune cell changes over the course of treatment. Most strong or moderate correlations with radiotherapy planning parameters were found at the early time point 4 weeks into radiotherapy (B/A), while only one moderate correlation occurred at the end of radiotherapy (data not shown).

### Correlation between T cell ratios and radiotherapy planning volumes

**Fig. 5:**
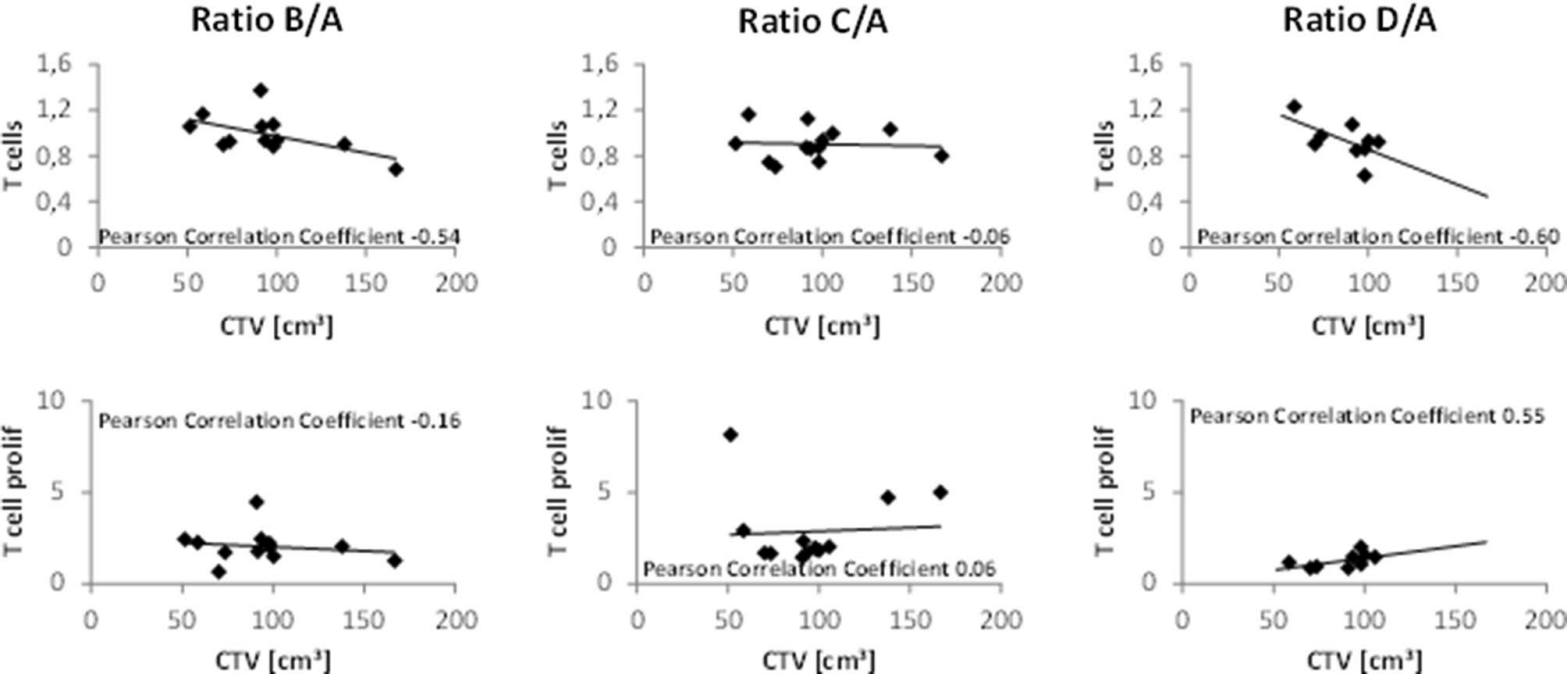
Changes of T cells and T cell proliferation are shown for time points B/A, C/A and D/A in the primary analysis of patients receiving radiotherapy to prostate only. T cells negatively correlated with CTV volume when comparing time points B and D, but not C, to A. T cell proliferation was positively correlated with CTV volume for ratio D/A, while having the lowest total ratio at this time point

The T cell ratios showed a moderate negative correlation with CTV volume for time points B/A and D/A, while no correlation was found for time points C/A (Fig. 5, upper panel). Ratio C/A was the only time point with a significant decrease in T cells compared to baseline (Fig. 1). T cell proliferation showed a moderate positive correlation with CTV volume for ratio D/A only (Fig. 5, lower panel), while the absolute values of T cell proliferation were the lowest at this time point and did not differ significantly from baseline. For patients receiving pelvic nodal irradiation (data not shown), for which an exploratory analysis was conducted, T cell ratios differed to those of patients treated to the prostate only, showing a strong positive correlation with CTV volume for time point ratios B/A and C/A (0.86 and 0.89, respectively). For ratio D/A, however, a moderate negative correlation was found (-0.45). Again, T cell proliferation only showed a correlation for ratio D/A, although here, a moderate negative correlation with CTV volume could be observed (-0.52). These data do not confirm the results of the primary analysis.

### Correlation between CD8^+^ effector cells and Treg cells and radiotherapy planning volumes

**Fig. 6:**
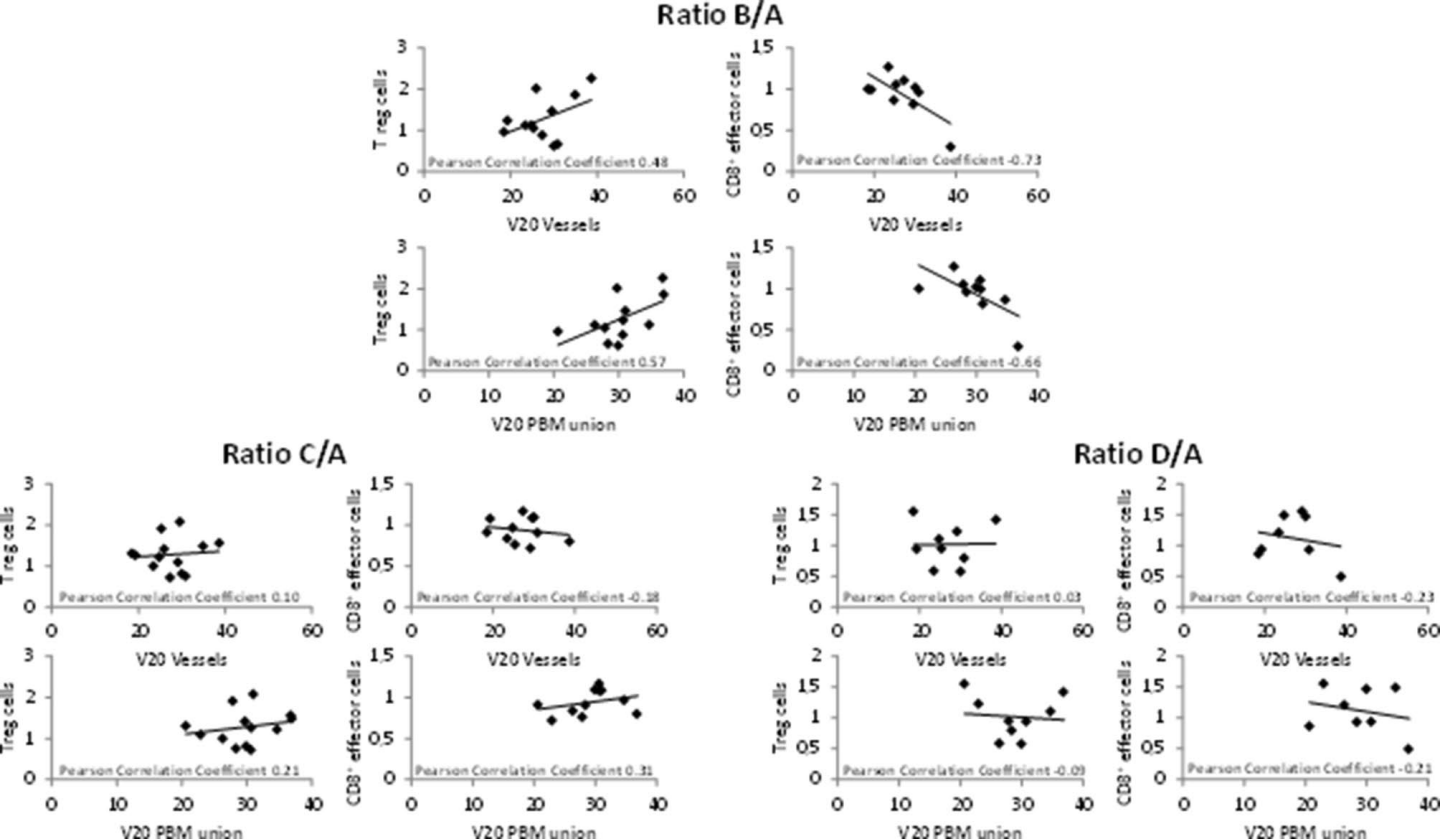
Ratios of Treg cells as well as CD8^+^ effector cells are shown in correlation with V20 (blood vessels) and V20 (PBM union) for time points B/A, C/A and D/A. Treg cells correlated positively with V20 (blood vessels) as well as V20 (PBM) at time point B/A in the primary analysis. At the same time, CD8^+^ effector cell ratios showed a negative correlation with V20 (blood vessels) as well as V20 (PBM). No such correlation was found with the ratios at time points C/A or D/A

Early changes (B/A) in CD8^+^ effector cells, both not significantly different from baseline, showed a moderate negative correlation with V20 (vessels) as well as a strong negative correlation with V20 (PBM) (Pearson coefficients: − 0.66 and − 0.73, respectively). At the same time, Treg cell ratios showed moderate positive correlations with both volume parameters (Fig. 6, upper panels). No correlation was found at later time points at the end of radiotherapy or 3 months thereafter (Fig. 6, lower panels). An exploratory analysis of the five patients who underwent radiotherapy to the prostate as well as pelvic nodal regions hints at a confirmation of those results with positive correlations of V20 (vessels) and V20 (PBM) with Treg cells (Pearson correlation coefficient 0.80 and 0.93, 0.90 and 0.94, 0.87 and 0.74 for ratios B/A, C/A and D/A, respectively). The negative correlation of V20 (vessels) and V20 (PBM) with CD8^+^ effector cells was confirmed as well for time points during treatment (Pearson correlation coefficient -0.74 and -0.99, -0.70 and -0.84, -0.42 and -0.30 for ratios B/A, C/A and D/A, respectively, data not shown).

## Discussion

We demonstrated previously that peripheral immune cell subsets are affected during and after curative, normofractionated radiotherapy for intermediate to high-risk prostate cancer [21]. Here, we show that these changes are associated with patient age, target volume and especially with contoured immune-related volumes (bone marrow and blood vessels). These correlations are mostly observed early during treatment (4 weeks into radiotherapy as compared to levels before start of the treatment), and persist 3 months after end of treatment.

This small study included patients with localized, node-negative prostate cancer. Especially for patients treated with elective nodal radiation, no conclusions can be drawn from our data as the sample size is limited to five patients. In addition, clinical variables such as T stage and PSA as well as treatment concepts differed significantly. For the performed analysis, patients treated with radiotherapy to prostate with or without seminal vesicles were included in the primary analysis. Patients treated with additional elective nodal irradiation were analyzed separately in an exploratory analysis. Two patients with intermediate-risk prostate cancer declined the use of ADT in addition to radiotherapy, which might influence the immune status of the patients [33]. However, these patients were included in the primary analysis. CTV volume and thus possibly dose to immune-related volume might differ for patients with T3b tumors with inclusion of the whole seminal vesicles into the CTV.

It is well established that aging is associated with alterations of the immune system, especially in the T cell compartment. Frequencies of naïve CD4^+^ and CD8^+^ T cells decrease with age in healthy individuals, while effector and memory subsets increase. In addition, T cell function is often impaired, in particular, in CMV positive individuals [34]. Such changes are thought to have an essential impact on prophylactic vaccination, but also on therapeutic manipulations of the immune system. As prostate cancer is mostly a disease of the elderly, these considerations are substantial. In animal models, T cells from young mice are more effective at eliminating tumors than these of older mice [35]. In head and neck squamous cell xenografts, tumor growth was also shown to be more rapid in old mice compared to young ones. However, upon checkpoint inhibition, tumors of older mice responded better, and this was associated with higher expression levels of immune checkpoint molecules CTLA-4 and PD-1 on T cells [36]. Similar observations have been made in some reports on melanoma patients treated with immune checkpoint inhibition [37].

The contouring strategy for pelvic bone marrow described by Li et al. [31] was confirmed. A significant difference between bone marrow and bone delineation was not found. Bone delineation is possible with semi-automatic contouring on CT only datasets such as planning CTs for radiotherapy [38]. In addition, since circulating lymphocytes have been described as being a “moving organ at risk” for radiotherapy [39, 40], large pelvic vessels were contoured. It has been recently proposed that immunotherapy combined to “lymphocyte sparing radiotherapy” could lead to improved clinical responses [41]. Vessel and bone marrow volumes were only partly associated with each other and with the volume of the CTV in our prostate carcinoma patient cohort, so that all three parameters were used for further analysis.

The volume of the CTV was inversely associated with changes of total T cells early during radiotherapy, as well as in the recovery phase after end of treatment. Sage et al. described a decrease in T cells at the end of radiotherapy for prostate cancer patients. However, they did not investigate associations with CTV volumes or other radiotherapy planning parameters [42].

During radiotherapy, large volumes of bone marrow and vessels receiving radiation doses > 20 Gy were associated with higher ratios of Treg cells and lower ratios of CD8^+^ effector cells. These data suggest an early impairment of anti-tumor immunity with larger irradiated immune-related volumes and go along with the known radioresistance of Tregs. Treg cells are major immunosuppressive players in the context of tumors, and normofractionated radiotherapy has been described to lead to an accumulation of Treg cells [43]. Immunohistochemical staining of the Treg cell marker FoxP3 following neoadjuvant radiochemotherapy for esophageal cancer has been reported as a prognostic marker for cancer-specific survival [44]. In contrast, CD8^+^ effector cells are the main effectors of anti-tumor immune response (reviewed in [45]). For instance, in patients receiving stereotactic radiotherapy for oligometastatic prostate cancer, an increase in tumor-reactive CD8^+^ effector cells was correlated with local disease control [46]. Whereas irradiation can promote T cell activation via dendritic cells in preclinical tumor models in vivo [47], patients treated with radiochemotherapy for cervical cancer showed significant reduction of CD8^+^ T cells during treatment [19]. Cytotoxic T cells have been linked to the outcome of patients with several cancers, e.g., anal cancer treated with radiochemotherapy [48]. Combinatorial approaches of radiotherapy and immunotherapy seem to be dependent on activation of CD8^+^ effector cells via dendritic cells [12]. Thus, the increase in Tregs and decrease in CD8^+^ effector cells with larger volumes of immune-related structures receiving radiation doses > 20 Gy at an early time point during treatment might reflect a transient impairment of the anti-tumor immunity. Irradiation of large volumes, especially elective nodal regions, has received a lot of attention concerning possible negative effects on anti-cancer immunity, so new concepts might emerge for node positive prostate cancer, especially in settings combining radiotherapy with immunotherapy [49]. These hypotheses might also form the basis on limiting the target volume margins in prostate radiotherapy for deliberate sparing of the obturator region.

Pelvic bone marrow volumes were already used to generate dosimetric data associated with acute hematologic toxicity during radio(chemo)therapy of pelvic malignancies, mostly rectal or anal cancer [50–55]. Those findings have led to the concept of bone marrow sparing radiotherapy to avoid these toxicities [52, 56, 57]. However, experimental studies did not differentiate immune cell subsets in comparable depth to our study. In addition, sparing of bone marrow might lead to higher radiation doses in other immune-related pelvic volumes.

### Conclusions

In conclusion, especially early changes of peripheral immune status during radiotherapy as well as long-lasting changes during the recovery phase appear to correlate with radiotherapy planning volumes and irradiated volumes of immune-associated pelvine structures in definitive treatment of prostate cancer. Maximal changes at the end of treatment seem to be mostly independent of radiotherapy planning parameters in this clinical setting. In order to further characterize the dependence of immune changes during treatment with radiotherapy planning parameters, early and late time points should be included in future analyses. Early impairment of anti-tumor immunity with larger irradiated volumes might provide a rationale for actively sparing immune-related volumes during prostate cancer radiotherapy, especially in combinatorial approaches with immunotherapy strategies.

## Supplementary Information

Below is the link to the electronic supplementary material.

Supplementary file1 (DOCX 12 kb)

Supplementary file2 (PDF 118 kb)

## Data Availability

Research data will be shared upon request.
