# Supplementary material for: Radiotherapy planning parameters correlate with changes in the peripheral immune status of patients undergoing curative radiotherapy for localized prostate cancer
Source: Cancer Immunol Immunother. 2021 Jul 16;71(3):541–52. doi: 10.1007/s00262-021-03002-6 (PMC8854140; doi:10.1007/s00262-021-03002-6)
Supplement: Supplementary file 1 — Supplementary file1 (DOCX 12 kb) [file 262_2021_3002_MOESM1_ESM.docx]

***Suppl. Table 1.*** For all analyzed cell subsets at all time points, numbers of available samples are stated for the primary analysis. Numbers vary due to samples which did not pass quality assurance of flow cytometry analysis (e.g. low cell numbers for small subpopulations).

***Suppl. Table 2.*** Pearson Correlation Coefficients are displayed for all immune cell subsets (ratios time points B, C and D to time point A) and analyzed clinical and radiotherapy planning parameters. For a more comprehensive overview, correlations for several volume parameters are stated (V10, V20, and V30 for vessels and V10, V20, V30, V40, and V50 for PBM union). V40 and V50 are not reported for vessels, as in many cases these dose levels were not reached in these volumes.
