# Supplementary material for: Radiotherapy planning parameters correlate with changes in the peripheral immune status of patients undergoing curative radiotherapy for localized prostate cancer
Source: Cancer Immunol Immunother. 2021 Jul 16;71(3):541–52. doi: 10.1007/s00262-021-03002-6 (PMC8854140; doi:10.1007/s00262-021-03002-6)
Supplement: Supplementary file 2 — Supplementary file2 (PDF 118 kb) [file 262_2021_3002_MOESM2_ESM.pdf]

Supplemental Tables

Suppl. Table 1.

Patient numbers										
**Time point B/A**	T cells	12		**Time point C/A**	T cells	13		**Time point D/A**	T cells	9
	CD8	12			CD8	13			CD8	9
	CD4	12			CD4	13			CD4	9
	Tregs	12			Tregs	13			Tregs	9
	B cells	12			B cells	13			B cells	9
	NK cells	12			NK cells	13			NK cells	9
	T-Zell prolif	12			T-Zell prolif	13			T-Zell prolif	9
	CD8 prolif	11			CD8 prolif	13			CD8 prolif	9
	CD4 prolif	12			CD4 prolif	13			CD4 prolif	9
	Treg prolif	12			Treg prolif	13			Treg prolif	9
	B-Zell prolif	12			B-Zell prolif	13			B-Zell prolif	9
	NK prolif	12			NK prolif	13			NK prolif	9
	Naive CD4	10			Naive CD4	11			Naive CD4	8
	Effector CD4	10			Effector CD4	11			Effector CD4	8
	Naive CD8	10			Naive CD8	11			Naive CD8	8
	Effector CD8	10			Effector CD8	11			Effector CD8	8
	Memory CD8	11			Memory CD8	11			Memory CD8	8

Suppl. Table 2.

Time point B/A

			Vessels			PBM union				
	Age	CTV	V10	V20	V30	V10	V20	V30	V40	V50
T cells	-0,43685851	-0,54927658	0,25766515	0,32876894	0,25074663	0,25931437	0,35073119	0,223862	0,14872298	0,14098339
CD8 pos	0,02425803	0,43126091	-0,45338241	-0,57482781	-0,21059821	-0,43805271	-0,33393627	-0,24068222	-0,17042493	-0,10317518
CD4 pos	-0,0731589	-0,45798232	0,26840757	0,28487492	-0,11095252	0,22571346	0,11287333	-0,02007366	-0,12402249	-0,1946782
Tregs	-0,15115149	-0,1680474	0,25675212	0,45839766	0,4762981	0,47492086	0,56637526	0,48316667	0,48601571	0,44726329
B cells	-0,28753678	-0,41634368	0,036403	0,0139421	-0,24396726	-0,03076374	-0,01716306	-0,17329708	-0,19370599	-0,20271397
NK cells	0,4327795	0,58542058	-0,16014038	-0,16158712	0,01294459	-0,08510035	-0,21872459	-0,01451875	0,05310102	-0,03334413
prolif T cells	-0,42248263	-0,15537616	0,35791761	0,44694751	0,48024855	0,39270759	0,52510005	0,44973687	0,38633608	0,30575749
prolif CD8 pos	-0,08499114	-0,10929196	0,03629764	0,17768291	-0,2099157	0,32199405	0,46541904	0,34227207	0,09269791	0,05516853
prolif CD4 pos	-0,40938601	-0,01821935	0,15447004	0,22040421	0,35699271	0,22228415	0,40316046	0,26317545	0,21936265	0,22264558
prolif Tregs	0,13605696	0,07842954	-0,00227184	-0,06817309	-0,10304829	-0,15320517	-0,15598678	-0,20116204	-0,18858341	-0,16691625
prolif B cells	-0,20925307	0,09207637	0,37656415	0,38058351	0,40531704	0,34283288	0,28240658	0,28168592	0,1609311	0,08228174
prolif NK cells	-0,24998002	-0,08191232	0,3169939	0,48908654	0,50859987	0,41624919	0,51468685	0,41389747	0,37817451	0,33988465
Naive CD4 pos	0,51446059	0,65762402	-0,20537836	-0,137959	0,04888799	-0,06366192	-0,27215205	-0,19886118	-0,0733067	-0,13662031
Effector CD4 pos	0,17008233	0,28966007	-0,18636516	-0,14932925	-0,24152792	-0,03494961	-0,01673438	-0,17133951	-0,28349143	-0,19481035
Naive CD8 pos	0,44762736	0,46745296	0,03403896	0,3621661	0,54455264	0,45371318	0,20666839	0,2191849	0,35260041	0,35486488
Effector CD8 pos	0,46076803	0,15039493	-0,55330447	-0,69401069	-0,72891807	-0,57263644	-0,65711875	-0,68664023	-0,68936019	-0,6638852
Memory CD8 pos	-0,61901349	-0,1821987	0,44377799	0,50834807	0,60022425	0,38554394	0,53186523	0,48729559	0,49307424	0,51957651

Time point C/A

			Vessels			PBM union				
	Age	CTV	V10	V20	V30	V10	V20	V30	V40	V50
T cells	0,2613306	-0,08177062	-0,24190305	0,11383654	0,05302082	0,28582581	0,42860933	0,19163288	0,22436778	0,43879749
CD8 pos	-0,14844792	-0,0821307	-0,10302299	-0,11959277	-0,00934581	-0,21455569	-0,05912422	-0,04046072	0,12086601	0,29742982
CD4 pos	-0,17820198	-0,21209773	0,16475808	0,11982349	-0,11781838	0,12265705	0,0001046	-0,05258136	-0,22140455	-0,40358604
Tregs	0,11285001	-0,40159704	-0,07422175	0,10813959	0,19087313	0,13420225	0,17238864	0,11739019	0,15609121	0,21692429
B cells	-0,37696723	-0,15593805	0,33074923	-0,21289573	-0,11883879	-0,61112404	-0,62595935	-0,43889799	-0,33184316	-0,37516074
NK cells	-0,12257783	0,06630362	-0,10923247	-0,18480717	-0,06475021	-0,15914164	-0,28876879	-0,05156845	-0,05974944	-0,22957323
prolif T cells	0,29900794	0,07597552	-0,04211854	0,01715867	-0,08148918	0,16921528	0,16059337	0,07787112	0,03512797	-0,0162122
prolif CD8 pos	0,32373504	0,41612644	0,11078154	0,17814993	0,204595	0,3167669	0,24373646	0,23860167	0,21061572	0,0950005
prolif CD4 pos	0,43946556	0,17435118	-0,1167258	-0,01017747	-0,0322086	0,17925062	0,18238487	0,09243787	0,07946137	0,07418462
prolif Tregs	0,55188235	0,16869848	-0,21441897	0,00049155	-0,02281357	0,20855778	0,27607064	0,16622381	0,21146169	0,3481288
prolif B cells	0,36325639	0,36270589	-0,13627623	-0,00797255	0,15802281	0,19628522	0,14626713	0,09740709	0,08766672	-0,00092052
prolif NK cells	0,30809406	0,10112099	-0,05271996	0,18034218	-0,04143574	0,29609174	0,40406072	0,20754792	0,21604186	0,40615398
Naive CD4 pos	-0,35353535	-0,13972767	0,47199931	0,55885443	0,42604355	0,43599194	0,32606558	0,46904224	0,41181548	0,26921796
Effector CD4 pos	0,03294606	0,02857346	0,20988627	0,14298501	-0,16663201	0,13671133	0,00202575	-0,08737682	-0,2945957	-0,3225229
Naive CD8 pos	0,3695626	-0,18157365	-0,03660034	0,19215002	0,29473827	0,30536916	0,15053204	0,24062839	0,24646011	0,17437252
Effector CD8 pos	-0,01014191	0,45341477	-0,18935345	-0,13809955	-0,02847806	0,08575548	0,1248291	-0,01985047	-0,12120537	-0,0383473
Memory CD8 pos	-0,21307134	-0,34254725	0,35915082	-0,01493092	-0,05266924	-0,34966565	-0,42095742	-0,21350247	-0,13138475	-0,32017503

Time point D/A

			Vessels			PBM union				
	Age	CTV	V10	V20	V30	V10	V20	V30	V40	V50
T cells	-0,51505986	-0,60320342	-0,22972119	-0,17602347	0,18286822	-0,08941995	0,11489261	0,07399073	0,20426529	0,31918926
CD8 pos	-0,37673048	0,22214081	0,232398	-0,09185435	-0,28341887	-0,42407973	-0,34031641	-0,22837593	-0,15999042	-0,16890688
CD4 pos	0,30866078	0,0395459	0,1630374	0,31769689	0,39770374	0,37300347	0,18753777	0,15299714	0,10791153	0,03698517
Tregs	-0,71198355	-0,23035178	0,25608695	0,02578691	0,38509381	-0,25478162	-0,09322154	0,08804298	0,1825975	0,08728861
B cells	-0,3698783	0,26614869	0,60916088	0,23492668	-0,01056602	-0,26837204	-0,34995088	-0,25233213	-0,09559902	-0,12117557
NK cells	0,68203632	0,41866227	-0,01060201	0,12333644	-0,08633923	0,2720421	0,13055586	0,21735625	0,10081211	-0,02601528
prolif T cells	0,21745869	0,54650143	0,11454715	0,17763322	-0,26576943	0,11130926	0,04900734	0,00209022	-0,14495787	-0,11082609
prolif CD8 pos	0,25006115	0,33729983	0,140971	0,33359832	-0,0138323	0,29533761	0,17781655	0,13110994	0,0017187	0,04472281
prolif CD4 pos	0,13273902	0,40147785	-0,09518848	-0,01409174	-0,3424188	0,08450749	0,16164879	0,15340281	0,01715991	0,05041127
prolif Tregs	0,26926073	0,28245306	-0,0603397	0,08936716	-0,24063554	0,40743341	0,41830871	0,37807086	0,18113805	0,18243583
prolif B cells	0,41678779	0,06636008	-0,24983914	0,10843968	0,06247519	0,51023441	0,51160623	0,49009263	0,27981775	0,25341734
prolif NK cells	-0,12838682	0,20857535	-0,20981122	-0,02996999	-0,13035063	0,15237182	0,36624717	0,30330451	0,24497944	0,34368374
Naive CD4 pos	0,63701261	0,66330353	-0,0858266	0,18238409	0,00841936	-0,16378737	-0,27269904	-0,27034124	-0,18980513	-0,12478047
Effector CD4 pos	0,29978901	0,22831528	0,09985133	0,06392049	-0,30211497	0,2163181	0,15936765	0,21347698	0,16289537	0,13579293
Naive CD8 pos	-0,07198493	0,02089217	-0,18498078	-0,2100735	-0,03858392	-0,57444512	-0,55436406	-0,55740863	-0,47137981	-0,41468676
Effector CD8 pos	0,66600819	0,45806866	-0,40147429	-0,20531159	-0,42417593	-0,22492303	-0,23497639	-0,23165844	-0,31790882	-0,27784501
Memory CD8 pos	-0,74884005	-0,72499459	0,15826628	-0,00159683	0,26303077	0,19566641	0,28319086	0,23940813	0,25879747	0,26099906
